# Burden of Food-Borne Trematodiases in China: Trends from 1990 to 2021 and Projections to 2035

**DOI:** 10.3390/tropicalmed9120295

**Published:** 2024-12-03

**Authors:** Yanzheng Zou, Yihu Lin, Yili Qian, Luqiu Tao, Gao Tan, Hongru Zhu, Li Pan, Xiaoli Liu, Yu He, Wei Wang

**Affiliations:** 1 National Health Commission Key Laboratory of Parasitic Diseases Prevention and Control, Wuxi 214064, China; zouyanzheng@jipd.com (Y.Z.); qianyili@jipd.com (Y.Q.); taoluqiu@jipd.com (L.T.); zhuhongru@jipd.com (H.Z.); panli@jipd.com (L.P.); 2 Jiangsu Provincial Key Laboratory on Parasites and Vector Control Technology, Wuxi 214064, China; 3 Jiangsu Institute of Parasitic Diseases, Wuxi 214064, China; 4 Quanzhou Woman’s and Children’s Hospital, Quanzhou 362000, China; a15960750926@163.com; 5 Hospital of Hunan Provincial Corps of the Chinese People’s Armed Police Force, Changsha 410006, China; ttgg_123@126.com (G.T.); liuxiaoli515@126.com (X.L.); 6 Chinese Preventive Medicine Association, Beijing 100062, China

**Keywords:** food-borne trematodiases, global burden of disease, China, disability-adjusted life years, prevalence

## Abstract

To assess the burden of food-borne trematodiases in China from 1990 to 2021 and project the burden through 2035, data were captured from the Global Burden of Disease Study (GBD) 2021 datasets. The estimated prevalent food-borne trematodiase cases were 33.32 million (95% uncertainty interval (*UI*): 29.25–38.35 million) in China in 2021, contributing to 768,297.4 disability-adjusted life years (DALYs) (95% *UI*: 383,882.8–1,367,826.1). The number of prevalent cases and DALYs declined by 9.02% and 18.11%, and a downward decline was seen in age-standardized prevalence and DALY rates (estimated annual percentage change: −0.96% and −1.21%, respectively). A higher prevalence and DALY rates were observed among males than females, and the middle-aged group bore the highest burden, while the older population showed the most rapid increase in prevalent cases and DALY numbers. Projected DALY counts and rates remain stable through 2035 using the Bayesian age–period–cohort (BAPC) model. These findings demonstrate a decline in the burden of food-borne trematodiases in China from 1990 to 2021; however, the prevalence remained high, which contributed considerably to disability and premature death. Continued control efforts and targeted interventions are essential to further reducing the burden of food-borne trematodiases in China.

## 1. Introduction

Food-borne trematodiases are infections caused by trematodes transmitted to humans primarily through the consumption of raw or undercooked fish, crustaceans, or aquatic plants [1,2]. These trematodes are classified into liver, lung, or intestinal flukes based on the organs they target in the host [1,2]. Although over 80 species of food-borne trematodes have been identified to cause human infections, only a few are of public health importance [3]. The World Health Organization (WHO) has identified four genera with the highest public health burdens, including *Clonorchis*, *Fasciola* and *Opisthorchis* (liver flukes) and *Paragonimus* (a lung fluke) [4].

Infections with food-borne trematodiases are often asymptomatic, and when symptoms do occur, they are usually non-specific [5]. Current diagnostic techniques rely largely on microscopic detection of eggs in feces, sputum, or other biofluids, which is of suboptimal effectiveness and incapable of species-specific distinction [6,7]. These diagnostic limitations, along with the wide range of trematode species and the often non-specific symptoms, lead to the underestimation of the burden of food-borne trematodiases, which continue to be classified as neglected tropical diseases (NTDs). Although neglected, food-borne trematodiases can lead to serious consequences, including cholangiocarcinoma and ectopic infections [2]. According to the WHO Foodborne Disease Burden Epidemiology Reference Group (2015), the four above-mentioned genera of food-borne trematodes collectively account for over 2 million disability-adjusted life years (DALYs) globally [8]. Currently, food-borne trematodiases are targeted for control in the WHO *NTD Road Map 2021–2030*, which aims to reduce the incidence, prevalence, morbidity, and/or mortality of food-borne trematodiases to a locally acceptable level and to maintain this reduction through continued efforts [9].

Food-borne trematodiases have a long history in China, with documented cases dating back several thousand years [10]. China has conducted three national surveys during the last forty years on the status of important human parasitic diseases, covering the periods 1988–1992, 2001–2004, and 2014–2016. Across these surveys, the infection rate of *Clonorchis sinensis* demonstrated a fluctuating trend: it increased from 0.31% in the first survey to 0.58% in the second, and then declined to 0.47% in the third. Meanwhile, the infection rates of *Paragonimus* and *Fasciola hepatica* decreased constantly up to the third survey [11,12,13]. Unfortunately, no national survey has been conducted since then, leaving a gap in national data after 2016. Additionally, the surveys did not cover the full spectrum of food-borne trematodiases nor assess the disease burden in terms of DALYs.

The Global Burden of Disease Study (GBD) provides a reliable and comprehensive database for examining the burden of various health conditions worldwide [14,15]. Based on the latest GBD 2021 database, the current study evaluated the prevalence and DALYs of food-borne trematodiases in China from 1990 to 2021, and projected the DALYs of this disease in China through 2035.

## 2. Methods

### 2.1. Data Source

Data on the prevalence and DALYs of food-borne trematodiases in China were obtained from GBD 2021 data resources, via the Global Data Health Exchange (GHDx) online platform [16]. GBD 2021 data resources provide a comprehensive epidemiological assessment of the burden associated with 371 diseases and injuries across 204 countries and territories. The detailed methodological framework and data sources for GBD 2021 have been detailed in the previous literature [14,15].

The GBD study employed Disease Modelling Meta-Regression version 2.1 (DisMod-MR 2.1) for the estimation of prevalence. DisMod-MR 2.1 is a Bayesian meta-regression tool that generates internally consistent estimates of prevalence by sex, location, year, and age group. This model accounts for variations in study design and methodology across diverse data sources, ensuring the consistency and accuracy of estimates [17].

Chinese data were primarily collected from the national population census, the disease surveillance system, the maternal and child health surveillance system, the cause of death reporting system of the Chinese Center for Disease Control and Prevention, the cancer registry system, and relevant published studies. Food-borne trematodiase cases were identified using the International Classification of Diseases (ICD) codes: ICD-9 (121–121.9, V75.6) and ICD-10 (B66–B66.9, B72.0). DALYs for food-borne trematodiases were calculated as the sum of years of life lost due to premature mortality (YLLs) and years lived with disability (YLDs) due to this disease.

All GBD estimates were reported with 95% uncertainty intervals (*UI*s), representing the 2.5th and 97.5th percentile values based on the distribution of 500 draws at each step of the GBD estimation process. These intervals reflect the uncertainty propagated through each stage of estimation. The age distribution of the world population from GBD 2021 was applied for estimation of age-standardized prevalence and DALY rates [2].

### 2.2. Statistical Analysis

To quantify temporal trends in age-standardized and age-specific prevalence and DALY rates, the estimated annual percentage change (EAPC) was calculated using a linear regression model, where the natural logarithm of the value was regressed against the calendar year. The equation used was: *y* = α + β*x* + ε, where *y* represents the natural logarithm of the value, *x* indicates the calendar year, α is the intercept, β denotes the slope, and ε is the error term. The EAPC was then calculated as 100 × [exp(β) − 1], with its 95% confidence interval (*CI*) derived from the linear regression model.

An EAPC with the lower limit of the 95% *CI* above zero indicates a statistically significant upward trend, whereas that with its upper limit below zero signifies a downward trend. If the 95% *CI* includes zero, it suggests the lack of statistically significant change in the value of interest.

To predict the future trends of food-borne trematodiases in China from 2022 to 2035, a Bayesian age–period–cohort (BAPC) model was employed, which incorporates integrated nested Laplace approximations (INLAs) for full Bayesian inference [18]. Previous studies have shown that the BAPC model provides superior coverage and precision compared to alternative prediction methods [19,20,21,22]. Prior distributions for the age, period, and cohort effects were assumed to follow inverse gamma distributions. A second-order random walk was employed to model the age, period, and cohort effects. Population estimates from 1990 to 2035 were obtained from the United Nations Department of Economic and Social Affairs Population Division [23].

A two-tailed *p* value of <0.05 was considered statistically significant. All statistical analyses were performed with R software version 4.4.1 (R Core Team; Vienna, Austria) [24].

## 3. Results

### 3.1. Prevalence and DALYs of Food-Borne Trematodiases in China from 1990 to 2021

**Figure 1:**
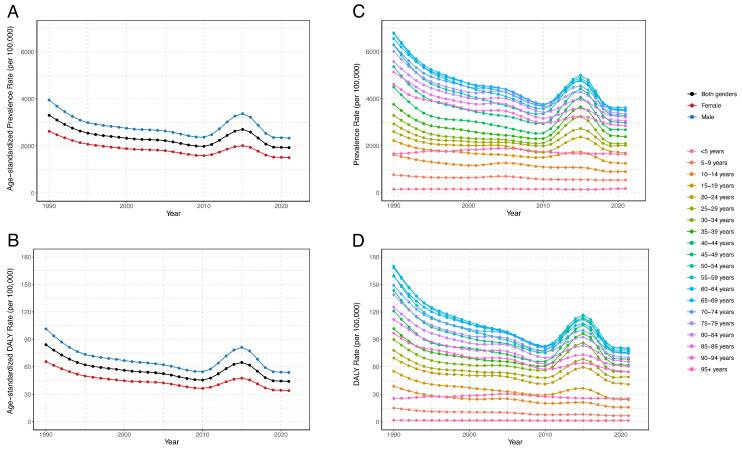
Trends in age-standardized and age-specific prevalence and DALY rates of food-borne trematodiases in China from 1990 to 2021. (**A**) Age-standardized prevalence. (**B**) Age-standardized DALY rates. The blue lines represent values for males, and the red lines represent values for females. These lines do not represent the same values but indicate gender-specific rates for the respective measures. (**C**) Age-specific prevalence. (**D**) Age-specific DALY rates. The meanings of the blue, red, and colored lines are described in the legends on the right side of the figures. Abbreviations: DALY, disability-adjusted life year.

There were an estimated 33,317,222.7 prevalent cases of food-borne trematodiases in China in 2021 (95% *UI*: 29,251,038.7 to 38,353,602.0), with an age-standardized prevalence of 1930.21 per 100,000 people (95% *UI*: 1700.47 to 2240.51 per 100,000 people). The prevalent cases decreased by 9.02% from 1990 to 2021, and the EAPC for the age-standardized prevalence was −0.96% (95% *CI*: −1.34% to −0.57%) (Table 1). The age-standardized prevalence showed a consistent downward trend from 1990 to 2010, followed by a brief increase from 2010 to 2015, before declining again up to 2021 (Figure 1A).

The estimated DALYs due to food-borne trematodiases were 768,297.4 (95% *UI*: 383,882.8 to 1,367,826.1) in China in 2021, with an age-standardized DALY rate of 44.17 per 100,000 people (95% *UI*: 22.08 to 79.87 per 100,000 people). The DALYs decreased by 18.11% from 1990 to 2021, and the EAPC for the age-standardized DALY rate was −1.21% (95% *CI*: −1.65% to −0.77%) (Table 2). Similarly to the trend in the prevalence rate, the age-standardized DALY rate increased from 2010 to 2015, followed by a continued decrease from 2015 to 2021 (Figure 1B).

In gender terms, both the number of prevalent cases and the age-standardized prevalence of food-borne trematodiases were higher among men males than among women from 1990 to 2021. Both genders exhibited a downward trend in the number and rate of prevalent cases over this period. A similar pattern was observed for DALYs, with men having higher DALY counts and age-standardized DALY rates than women, while both genders followed a downward trend (Table 1 and Table 2). Consistent with the overall trends, both males and females experienced a brief increase in age-standardized prevalence and DALY rates from 2010 to 2015, followed by a continued decline (Figure 1A,B).

### 3.2. Age-Specific Prevalence and DALYs of Food-Borne Trematodiases in China from 1990 to 2021

**Figure 2:**
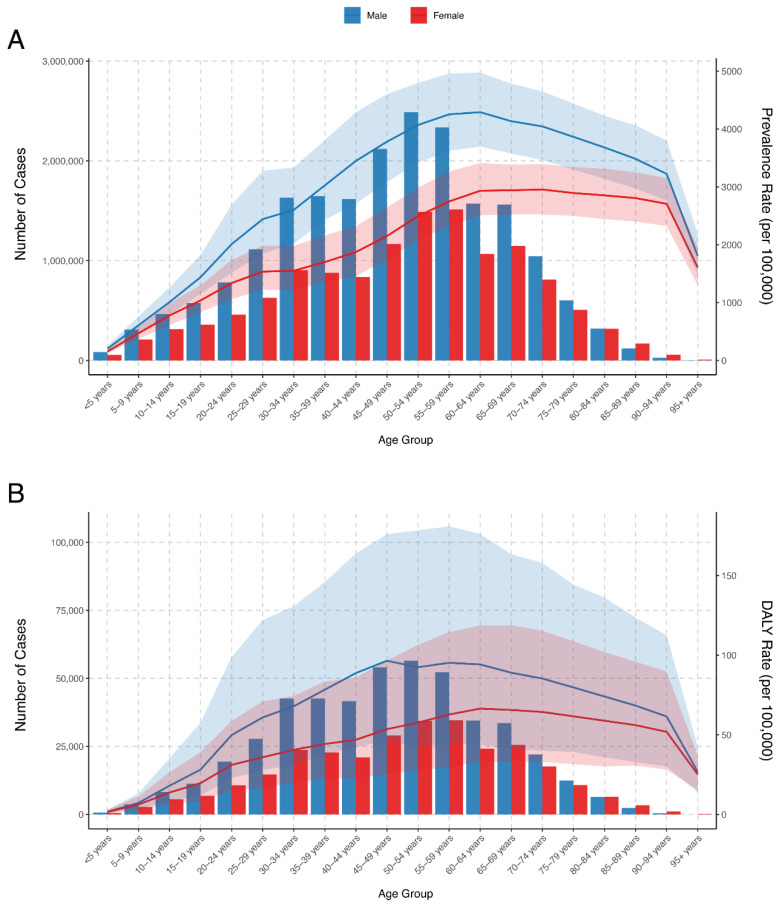
Age-specific prevalence and DALYs numbers and rates of food-borne trematodiases in China in 2021. (**A**) Prevalence (number of cases) and rates by age group and gender. (**B**) DALY numbers and rates by age group and gender. Abbreviations: DALYs, disability-adjusted life years. Blue lines indicate data among men, and red lines indicate data among women. The shadows indicate the 95% *CI*s for the prevalence and DALYs numbers and rates.

The prevalent cases of food-borne trematodiases increased with age in 2021, peaking in the 50–54-year age group (3,978,362.9, 95% *UI*: 3,342,141.3 to 4,704,312.9), and declining progressively with age. Across most age groups, the number of prevalent cases was higher among men than women, except in age groups above 85 years, where females had slightly higher case counts. The prevalence rate followed a similar pattern, increasing with age and peaking slightly later in the 60–64-year age group at 3613.95 per 100,000 population (95% *UI*: 3105.81 to 4203.59). After this peak, the prevalence rate declined in the older age groups. In terms of gender, males consistently showed higher prevalence rates across all age groups compared to females (Table S1, Figure 2A).

The prevalent cases of food-borne trematodiases decreased in younger age groups (under 45 years old) from 1990 to 2021 and increased in older age groups. The most significant growth occurred among people at ages of 95 years and older, with a percentage increase of 1465.37%. In contrast, the prevalence rate declined across most age groups during this period. The 10–14-year age group exhibited the largest decrease in the prevalence rate, with an EAPC of −1.46% (95% *CI*: −1.66% to −1.26%) (Table S1). Most age groups experienced an upward trend in prevalence rate after 2010, followed by a decline after 2015. However, this pattern did not apply to the very young (under 15 years old) and very old people (above 95 years old), where the rates remained relatively stable (Figure 1C).

The DALY counts due to food-borne trematodiases increased with age in 2021, peaking in the 50–54-year age group with 90,924.1 DALYs (95% *UI*: 43,634.0 to 175,210.7), before declining in older age groups. The DALY counts were higher among men than women across most age groups, although women surpassed men in DALY counts after the age of 80 years. Similarly, the DALY rate increased with age, reaching a peak in the 60–64-year age group at 80.35 per 100,000 population (95% UI: 39.32 to 144.57 per 100,000 population), before gradually declining. Men consistently exhibited higher DALY rates across all age groups compared to women (Table S2, Figure 2B).

DALY counts decreased in younger age groups (under 45 years) and increased in most older age groups from 1990 to 2021. The most significant growth was observed among people at ages of 95 years and older, with a percentage increase of 1464.42%. However, the DALY rate declined in most age groups during this period. The largest decrease in the DALY rate occurred in the 5–9-year age group, with an EAPC of −2.31% (95% *CI*: −2.51% to −2.10%) (Table S2). Similarly to the trend in prevalence rates, most age groups experienced a temporary increase in DALY rates after 2010, followed by a decline, except for the very young (under 15 years) and very old people (95 years and older), where DALY rates remained relatively stable (Figure 1D).

### 3.3. Projected DALYs of Food-Borne Trematodiases in China Through 2035

DALY counts due to food-borne trematodiases are projected to decrease in China by 2035, with an estimated DALY count of 636,726.2, representing a 17.13% reduction from 2021. In contrast, the DALY rate is expected to remain relatively stable through 2035. By 2035, the DALY rate is projected to be 46.10 per 100,000 population, reflecting only a modest increase from the rate in 2021.

**Figure 3:**
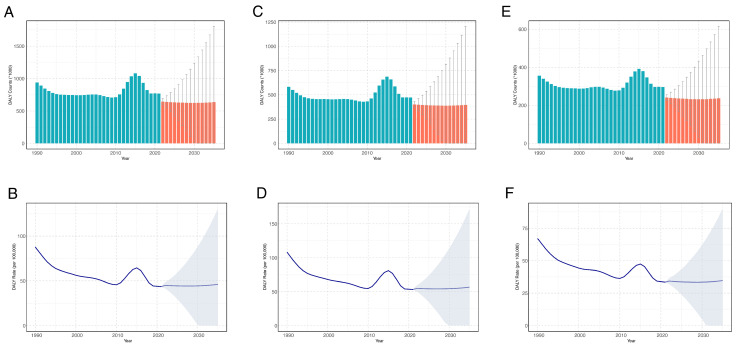
Prediction of age-standardized DALYs rates and counts for food-borne trematodiases in China from 2022 to 2035. (**A**,**B**) Both genders; (**C**,**D**) men; (**E**,**F**) women. The blue bars indicate observed values, and the red bars indicate predicted values. The shadows or error bars indicate the 95% *CI*s for the predicted rates and numbers. Abbreviations: DALY, disability-adjusted life years; *CI*, confidence interval.

In gender terms, men are expected to continue having higher DALY counts and rates of food-borne trematodiases than women by 2035. The DALY count for men is projected to be 396,135.5, while for men, it is projected to be 236,549.9, both representing decreases from 2021 levels. Meanwhile, the DALY rate is expected to be 56.69 per 100,000 population among men, and 34.66 per 100,000 population among women, with both rates remaining stable and showing no significant growth from 2021 (Figure 3).

## 4. Discussion

In this study, data from the GBD 2021 study were used to analyze the prevalence and DALYs of food-borne trematodiases in China from 1990 to 2021. Additionally, the BAPC model was employed to predict the DALYs of food-borne trematodiases through 2035.

In the current study, both the prevalent cases and DALY counts of food-borne trematodiases were found to decrease in China between 1990 and 2021. Similarly, the age-standardized prevalence and DALY rates showed an overall downward trend during this period. Several factors contributed to the reduction in the burden of food-borne trematodiases. First, China’s continuous social and economic development led to improved living standards, better infrastructure, and enhanced sanitation of food and drinking water [25]. Additionally, the implementation of integrated public health strategies, such as drug administration and information, education, and communication (IEC) campaigns, significantly reduced the disease burden [26,27]. A study evaluating community-based integrated strategies for clonorchiasis control in two pilot counties in China between 2007 and 2009 reported a sharp reduction in prevalence, from 41.4% to 7.0%, highlighting the effectiveness of these strategies [28].

However, despite the overall decline, the prevalence of food-borne trematodiases remains high in China and it continues to be a significant cause of disability and premature death. In 2021, 33,317,222.7 prevalent cases were estimated to suffer from food-borne trematodiases in China, contributing to 768,297.4 DALYs. The persistence of this burden is partly attributed to entrenched cultural practices in endemic areas, where the consumption of raw fish, crustaceans, and aquatic plants is common. These dietary habits are difficult to change, even with improved hygiene and ongoing health education. For instance, in the city of Shunde, Guangdong province, where raw fish sashimi is a traditional delicacy, an epidemiological survey during the period between 2014 and 2015 revealed that the prevalence of *Clonorchis sinensis* infections was as high as 42.38% among local residents [29]. Moreover, food-borne trematodiases have complex life cycles that involve multiple hosts, including intermediate hosts, reservoir hosts, and definitive hosts. The control of these non-human hosts is a great challenge and is resource-intensive [7].

The results from this study showed a brief increase in the prevalence and DALY rates of food-borne trematodiases after 2010, followed by a rapid decline after 2015. This decline is considered to be attributed to China’s National Plan for the Prevention and Control of Echinococcosis and Other Important Parasitic Diseases (2016–2020) [30]. The plan emphasized integrated preventive strategies for food-borne trematodiases, particularly liver flukes, including environmental management, IEC programs, and the deworming of high-risk populations. In addition, a national clonorchiasis surveillance system has been established since 2016, providing high-precision data on the epidemiology of the disease down to the county level [31]. This system has facilitated the early detection of clonorchiasis cases and has collected comprehensive epidemiological data, supporting long-term prevention and control efforts.

In gender terms, men consistently exhibited a higher prevalence and higher DALY rates of food-borne trematodiases than women, throughout the study period. This finding is consistent with an earlier systematic analysis of the global burden of human food-borne trematodiases, conducted as part of the GBD 2010 study and a WHO initiative [32]. The analysis demonstrated that the prevalence of most food-borne trematodiases was higher among men than among women, except for fascioliasis, where slightly more women were infected [32]. Furthermore, the 2014–2016 national survey on the status of important human parasitic diseases in China (the third national survey), also reported that the prevalence of *C. sinensis* infections was higher among men than among women in both rural and urban areas [13]. The discrepancy in disease burden between genders may result from the fact that men consume more risky foods in certain communities or are more frequently engaged in activities such as fishing and fish farming, which increase exposure to contaminated water and food sources.

Regarding age-specific patterns, the disease burden due to food-borne trematodiases increased among younger individuals in China, peaked in middle-aged groups, and then declined in older age groups. Previous studies analyzing the DALYs of major parasitic diseases in China using GBD 2019 data reported that over 60% of the burden of food-borne trematodiases was concentrated in the 30–69-year age group [33]. Previous studies examining different classifications of food-borne trematodiases have shown that the prevalence of liver and lung fluke infections typically increases steadily until plateauing in middle-aged individuals [34,35]. Although data on intestinal fluke infections are limited, it is assumed they follow a similar pattern. The high disease burden in the middle-aged groups is believed to stem from the long lifespan of food-borne trematodiases and the likelihood of continuous reinfection over time [3]. Additionally, an increase in prevalent cases and DALY counts of food-borne trematodiases was observed in older age groups in this study, likely due to the aging of the patient population. Older individuals are more likely to experience complications related to food-borne trematodiases due to weaker immune systems, comorbidities, and delayed diagnosis. This increased burden in older populations poses challenges for the prevention and treatment of food-borne trematodiases.

Lastly, the DALY counts and rates of food-borne trematodiases were projected in China through 2035. The DALY counts of food-borne trematodiases were expected to decrease, while the DALY rates were projected to remain stable with a slight increase. These projections suggest that controlling food-borne trematodiases remains a significant challenge in China. Encouragingly, China’s National Comprehensive Implementation Plan for the Prevention and Control of Echinococcosis and Other Important Parasitic Diseases (2024–2030) was issued in 2024 [36]. Building on the 2016–2020 plan, this updated version places a greater emphasis on the prevention of food-borne parasitic diseases as a whole. It advocates for proactive identification and treatment of infected individuals through surveillance, screening, and regular hospital visits. It also addresses non-human hosts by recommending regular deworming of animals in endemic areas. The plan aims to reduce the infection rate of liver flukes by 5% by 2025 and 15% by 2030 in key endemic provinces of China. It also sets a goal to maintain control efforts over lung fluke infections. To achieve these goals, it is recommended that future control strategies for food-borne trematodiases consider implementing more culturally sensitive interventions in China, such as introducing culturally acceptable alternatives to raw aquatic products and developing locally tailored health education materials. Additionally, based on the findings of the present study, future preventive strategies may adopt gender- and age-specific measures, particularly for men and middle- to older-aged individuals. For example, targeted IEC programs may be implemented in male-dominated industries, such as fishing or food vending, while regular screening and early detection are provided for middle- to older-aged groups in endemic areas.

To the best of the authors’ knowledge, this study is the first to comprehensively analyze the disease burden of food-borne trematodiases in China using the latest GBD 2021 data, while also providing projections of the future disease burden through 2035. Nevertheless, the study acknowledges several limitations. First, the current study shares the weaknesses of other GBD studies. The GBD data are based on model fitting rather than real-world data, which introduces uncertainty into disease burden estimates. Although the GBD employs rigorous statistical methods to address these uncertainties, the results should be interpreted with caution. Second, the analysis of this study was conducted at the national level as provincial or municipal disease burdens were not available. Further studies are needed to examine the subnational disease burdens of food-borne trematodiases in China. Finally, while the BAPC model was used to project the future DALYs of food-borne trematodiases, it may not fully capture the impact of various influencing factors, as the projections rely solely on historical data and population estimates.

## 5. Conclusions

This study provides a systematic analysis of the prevalence and DALYs of food-borne trematodiases in China based on the latest GBD 2021 data. Although the overall burden showed a downward trend from 1990 to 2021, it remained substantial in 2021. Men experienced a higher disease burden than women. The middle-aged group bore the highest burden, while the older population saw the most rapid increase in prevalence cases and DALY counts. The projections indicate that the DALYs due to food-borne trematodiases are likely to remain stable in China until 2035. China has made significant progress in controlling food-borne trematodiases and reducing disease burden; however, sustained preventive and control efforts, including targeted interventions for high-risk groups, are needed to achieve the goals set out in China’s National Comprehensive Implementation Plan for the Prevention and Control of Echinococcosis and Other Important Parasitic Diseases (2024–2030) and the WHO NTD roadmap (2021–2030) [9]. This study provides valuable insights into public health planning and supports the development of more focused and effective interventional strategies.

## Figures and Tables

**Table 1:** Overview of prevalence of food-borne trematodiases in China in 1990 and 2021.

Gender	Number		Percentage Change (%)	Age-Standardized Rate (Per 100,000 Population)		EAPC (95% *CI*, %)
	1990 (95% *UI*)	2021 (95% *UI*)		1990 (95% *UI*)	2021 (95% *UI*)	
Both	36,621,225.1 (31,239,359.0, 43,237,071.8)	33,317,222.7 (29,251,038.7, 38,353,602.0)	−9.02	3307.89 (2845.59, 3875.81)	1930.21 (1700.47, 2240.51)	−0.96 (−1.34, −0.57)
Male	22,476,187.2 (19,095,640.6, 26,580,789.7)	20,418,481.9 (17,785,189.7, 23,654,614.6)	−9.16	3954.06 (3377.44, 4640.68)	2337.02 (2042.27, 2718.69)	−0.81 (−1.24, −0.38)
Female	14,145,037.9 (12,067,609.2, 16,774,017.0)	12,898,740.8 (11,350,526.9, 14,870,728.4)	−8.81	2626.23 (2255.27, 3066.96)	1506.51 (1328.58, 1743.02)	−1.17 (−1.48, −0.85)

Abbreviations: EAPC, estimated annual percentage change; *UI*, uncertainty interval; *CI*, confidence interval.

**Table 2:** Overview of DALYs of food-borne trematodiases in China in 1990 and 2021.

Gender	Number		Percentage Change (%)	Age-Standardized Rate (Per 100,000 Population)		EAPC (95% *CI*, %)
	1990 (95% *UI*)	2021 (95% *UI*)		1990 (95% *UI*)	2021 (95% *UI*)	
Both	938,172.2 (365,200.0, 1,876,414.7)	768,297.4 (383,882.8, 1,367,826.1)	−18.11	84.04 (32.98, 168.04)	44.17 (22.08, 79.87)	−1.21 (−1.65, −0.77)
Male	582,518.0 (224,509.7, 116,8057.0)	472,583.6 (231,736.8, 849,985.0)	−18.87	101.43 (39.26, 203.48)	53.80 (26.54, 98.11)	−1.08 (−1.57, −0.59)
Female	355,654.2 (137,444.6, 708,492.9)	295,713.8 (151,020.2, 519,060.0)	−16.85	65.63 (25.81, 129.90)	34.10 (17.27, 60.67)	−1.39 (−1.75, −1.04)

Abbreviations: DALYs, disability adjusted life years; EAPC, estimated annual percentage change; *UI*, uncertainty interval; *CI*, confidence interval.

## Data Availability

All data presented in this study are available upon request to the corresponding author.

## Supplementary Materials

The following supporting information can be downloaded at: https://www.mdpi.com/article/10.3390/tropicalmed9120295/s1. Table S1: Age-specific prevalence of food-borne trematodiases in China in 1990 and 2021. Table S2: Age-specific DALYs of food-borne trematodiases in China in 1990 and 2021.
