# Supplementary material for: Burden of Food-Borne Trematodiases in China: Trends from 1990 to 2021 and Projections to 2035
Source: Trop Med Infect Dis. 2024 Dec 3;9(12):295. doi: 10.3390/tropicalmed9120295 (PMC11679480; doi:10.3390/tropicalmed9120295)
Supplement: Supplementary file 1 [file tropicalmed-09-00295-s001.zip › tropicalmed-3334722-supplementary.pdf]

Supplementary Materials

The following supporting information can be downloaded from the journal website.

Table S1: Age-specific prevalence of food-borne trematodiases in China in 1990 and 2021.

Table S2: Age-specific DALYs of food-borne trematodiases in China in 1990 and 2021.

Table S1: Age-specific prevalence of food-borne trematodiases in China in 1990 and 2021.

Age group (years)	Number		Percentage change (%)	Prevalence rate (per 100 000 population)		EAPC (95% *CI*, %)
	1990 (95%*UI*)	2021 (95%*UI*)		1990 (95%*UI*)	2021 (95%*UI*)	
<5	171,914.5 (144,671.2, 208,443.7)	139,694.9 (114,532.1, 171,235.8)	-18.74	153.76 (129.39, 186.43)	179.86 (147.46, 220.47)	0.05 (-0.14, 0.24)
5-9	799,728.8 (615585.6, 1,010,084.7)	520,272.9 (418,052.3, 648,896.7)	-34.94	766.92 (590.33, 968.65)	543.25 (436.52, 677.55)	-0.99 (-1.17, -0.81)
10-14	1,653,399.4 (1,218,426.7, 2,149,169.6)	776,875.7 (610,532.1, 979,481.6)	-53.01	1616.35 (1,191.12, 2,101.01)	901.32 (708.33, 1,136.38)	-1.46 (-1.66, -1.26)
15-19	2,812,747.8 (2,142,971.6, 3,626,489.7)	936,264.0 (753,794.3, 1,173,989.0)	-66.71	2,220.62 (1,691.84, 2,863.05)	1,253.83 (1,009.47, 1,572.19)	-1.17 (-1.44, -0.91)
20-24	3,436,931.0 (2,608,526.7, 4,458,216.2)	1,240,725.6 (955,563.4, 1,634,296.6)	-63.90	2,603.72 (1,976.14, 3,377.41)	1,695.58 (1,305.88, 2,233.43)	-0.63 (-0.98, -0.27)
25-29	3,219,883.5 (2,475,118.3, 4,211,276.7)	1,741,653.7 (1,345,850.6, 2,298,166.3)	-45.91	2,930.11 (2,252.37, 3,832.28)	2,013.89 (1556.22, 2,657.39)	-0.40 (-0.78, -0.02)
30-34	2,903,003.4 (2,268,678.5, 3,779,118.4)	2,538,187.6 (1,984,320.7, 3,241,193.0)	-12.57	3,289.73 (2,570.91, 4,282.56)	2,095.02 (1,637.86, 2,675.28)	-0.39 (-0.91, 0.13)
35-39	3,435,818.6 (2,709,447.3, 4,304,031.1)	2,523,380.2 (2,014,491.7, 3,165,399.0)	-26.56	3,761.61 (2,966.36, 4,712.15)	2,381.37 (1,901.12, 2,987.26)	-0.41 (-0.95, 0.13)
40-44	3,017,969.3 (2,405,475.4, 3,666,181.0)	2,454,304.9 (1,957,302.9, 3,031,967.6)	-18.68	4,498.10 (3,585.21, 5,464.22)	2,681.31 (2,138.34, 3,312.41)	-0.64 (-1.19, -0.08)
45-49	2,768,369.1 (2,274,417.5,	3,287,197.4 (2,690,789.9,	18.74	5,363.09 (4,406.17,	2,979.65 (2,439.04,	-0.93 (-1.44, -0.41)

	3,393,964.6)	4,014,219.7)		6,575.04)	3,638.65)	
50-54	3,001,413.2 (2,502,700.4, 3,644,828.9)	3,978,362.9 (3,342,141.3, 4,704,312.9)	32.55	6,290.84 (5,245.56, 7,639.41)	3,291.74 (2,765.32, 3,892.40)	-1.23 (-1.71, -0.75)
55-59	2,939,965.3 (2,472,520.4, 3,529,015.9)	3,847,655.0 (3,277,996.9, 4,521,053.7)	30.87	6,778.94 (5,701.11, 8,137.17)	3,499.70 (2,981.56, 4,112.20)	-1.37 (-1.81, -0.93)
60-64	2,403,019.1 (2,035,702.1, 2,875,610.9)	2,638,368.9 (2,267,403.2, 3,068,835.9)	9.79	6,800.20 (5,760.75, 8,137.57)	3,613.95 (3,105.81, 4,203.59)	-1.42 (-1.79, -1.06)
65-69	1,789,766.7 (1,518,589.8, 2,133,235.4)	2,706,401.5 (2,339,545.7, 3,102,609.6)	51.22	6,560.29 (5,566.31, 7,819.26)	3,528.39 (3,050.12, 4,044.94)	-1.41 (-1.72, -1.09)
70-74	1,185,961.6 (1,003,456.2, 1,409,151.9)	1,856,504.5 (1,589,514.4, 2,120,836.3)	56.54	6,302.39 (5,332.53, 7,488.46)	3,483.36 (2,982.40, 3,979.32)	-1.33 (-1.60, -1.07)
75-79	684,429.1 (580,074.5, 817,345.2)	1,109,850.9 (951,457.8, 1,267,878.7)	62.16	6,013.95 (5,097.00, 7,181.86)	3,351.11 (2,872.85, 3,828.26)	-1.31 (-1.56, -1.06)
80-84	295,455.9 (248,823.4, 352,100.8)	636,509.6 (542,502.1, 736,544.2)	115.43	5,577.66 (4,697.33, 6,647.02)	3,216.02 (2,741.04, 3,721.45)	-1.28 (-1.51, -1.05)
85-89	86,628.4 (72,630.0, 103,491.8)	290,944.6 (248,501.3, 338,972.4)	235.85	5,135.49 (4,305.64, 6,135.19)	3,054.30 (2,608.73, 3,558.48)	-1.32 (-1.53, -1.11)
90-94	14,148.4 (11,764.8, 17,181.7)	83,548.5 (71,718.7, 97,227.5)	490.52	4,611.27 (3,834.40, 5,599.90)	2,849.55 (2,446.08, 3,316.10)	-1.28 (-1.44, -1.11)
95 and older	672.0 (561.3, 792.9)	10,519.3 (8,546.3, 12,752.3)	1,465.37	1,659.66 (1,386.12, 1,958.21)	1,645.97 (1,337.24, 1,995.35)	-0.19 (-0.35, -0.02)

Abbreviations: EAPC, estimated annual percentage change; UI, uncertainty interval; CI, confidence interval.

Table S2: Age-specific DALYs of food-borne trematodiases in China in 1990 and 2021.

Age group (years)	Number		Percentage change (%)	DALY rate (per 100 000 population)		EAPC (95% *CI*，%)
	1990 (95%*UI*)	2021 (95%*UI*)		1990 (95%*UI*)	2021 (95%*UI*)	
<5	2,031.6 (1,081.8, 3,458.7)	1,227.1 (633.5, 1,972.2)	-39.60	1.82 (0.97, 3.09)	1.58 (0.82, 2.54)	-0.74 (-0.90, -0.57)
5-9	15,797.3 (6,328.7, 32,927.4)	6,441.4 (3,175.8, 11,675.0)	-59.22	15.15 (6.07, 31.58)	6.73 (3.32, 12.19)	-2.31 (-2.51, -2.10)
10-14	39,857.1 (14,192.0, 86,405.5)	13,801.5 (6,714.9, 26,475.6)	-65.37	38.96 (13.87, 84.47)	16.01 (7.79, 30.72)	-2.28 (-2.51, -2.05)
15-19	69,811.7 (24,070.1, 148,812.0)	18,072.2 (7,724.6, 37,470.3)	-74.11	55.12 (19.00, 117.48)	24.20 (10.34, 50.18)	-1.83 (-2.19, -1.47)
20-24	91,807.6 (34,189.3, 191,422.8)	30,048.7 (14,278.4, 58,379.7)	-67.27	69.55 (25.90, 145.02)	41.06 (19.51, 79.78)	-0.87 (-1.27, -0.47)
25-29	85,671.9 (31,447.9, 169,654.0)	42,446.5 (19,397.0, 87,010.0)	-50.45	77.96 (28.62, 154.39)	49.08 (22.43, 100.61)	-0.64 (-1.06, -0.22)
30-34	79,295.9 (31,337.0, 160,702.4)	66,289.6 (32,003.2, 125,248.6)	-16.40	89.86 (35.51, 182.11)	54.72 (26.42, 103.38)	-0.49 (-1.01, 0.04)
35-39	92,973.1 (36,213.0, 189,105.5)	65,380.4 (31,594.7, 123,636.0)	-29.68	101.79 (39.65, 207.04)	61.70 (29.82, 116.68)	-0.51 (-1.05, 0.04)
40-44	81,304.9 (30,671.0, 163,233.2)	62,564.1 (29,975.6, 114,785.1)	-23.05	121.18 (45.71, 243.29)	68.35 (32.75, 125.40)	-0.77 (-1.34, -0.21)
45-49	74,077.2 (27,619.0, 147,619.4)	83,120.4 (38,906.2, 150,098.9)	12.21	143.51 (53.51, 285.98)	75.34 (35.27, 136.06)	-1.08 (-1.60, -0.56)
50-54	76,218.4 (28,809.0, 157,441.2)	90,924.1 (43,634.0, 175,210.7)	19.29	159.75 (60.38, 329.99)	75.23 (36.10, 144.97)	-1.51 (-2.02, -1.00)
55-59	73,736.4 (27,983.8, 150,512.0)	86,778.8 (41,879.9, 162,705.9)	17.69	170.02 (64.52, 347.05)	78.93 (38.09, 147.99)	-1.65 (-2.11, -1.18)
60-64	59,478.0 (23,670.0, 119,142.2)	58,659.0 (28,705.5, 105542.6)	-1.38	168.31 (66.98, 337.16)	80.35 (39.32, 144.57)	-1.71 (-2.13, -1.30)
65-69	43,396.6 (17,667.0, 88,027.8)	59,120.3 (29,032.5, 107,203.1)	36.23	159.07 (64.76, 322.66)	77.08 (37.85, 139.76)	-1.69 (-2.05, -1.33)
70-74	28,064.7 (11,468.3, 56,576.6)	39,708.1 (19,789.4, 71,793.7)	41.49	149.14 (60.94, 300.66)	74.50 (37.13, 134.71)	-1.61 (-1.92, -1.30)
75-79	15,814.9 (6,682.0, 31,495.1)	23,228.6 (11,931.7, 4,0927.0)	46.88	138.96 (58.71, 276.74)	70.14 (36.03, 123.58)	-1.58 (-1.87, -1.29)
80-84	6,640.7 (2,915.3, 13,102.9)	12,959.5 (6,550.4, 22,750.2)	95.15	125.36 (55.04, 247.36)	65.48 (33.10, 114.95)	-1.54 (-1.81, -1.27)
85-89	1887.1 (822.7, 3588.0)	5,765.8 (3,041.6, 9,965.5)	205.54	111.87 (48.77, 212.70)	60.53 (31.93, 104.62)	-1.55 (-1.80, -1.31)
90-94	296.6 (134.6, 563.1)	1,598.4 (848.5, 2,809.7)	438.91	96.68 (43.85, 183.51)	54.52 (28.94, 95.83)	-1.49 (-1.69, -1.29)
95 and	10.4 (6.4, 15.0)	162.7 (101.1, 234.1)	1464.42	25.62 (15.90,	25.46 (15.81,	-0.19 (-0.40,

older	37.11)	36.64)	0.03)

Abbreviations: DALY, disability-adjusted life year; EAPC, estimated annual percentage change; *UI*, uncertainty interval; *CI*, confidence interval.
